# Research evolution of flat peach (*Prunus persica* (L.) Batsch): a decadal bibliometric analysis

**DOI:** 10.3389/fpls.2025.1710142

**Published:** 2025-11-28

**Authors:** Abebe Misganaw Gedamu, Yong Li, Jinlong Wu, Ke Cao, Muhammad Anees, Zhaohui Wang, Suning Liu, Hongyang Xing, Pengcheng Wei, Muhammad Jehangir, Umar Hayat, Wei Ren, Lirong Wang

**Affiliations:** 1 Zhengzhou Fruit Research Institute, National Key Laboratory for Germplasm Innovation & Utilization of Horticultural Crops, Chinese Academy of Agricultural Sciences, Zhengzhou, Henan, China; 2 College of Agriculture and Natural Resources, Mekdela Amba University, Tulu Awliya, Amhara, Ethiopia; 3 Institute of Western Agriculture, Chinese Academy of Agricultural Sciences, Changji, Xinjiang, China; 4 Zhongyuan Research Center, Chinese Academy of Agricultural Sciences, Xinxiang, Henan, China

**Keywords:** bibliometric, citation, fruit quality, flat peach, research landscape, structural variation

## Abstract

**Introduction:**

The flat peach (*Prunus persica* (L.) Batsch.), characterized by its oblate shape and determined by a known genetic inversion, is a high-value cultivar gaining global market share. Mapping the intellectual landscape of its research is crucial to consolidate knowledge and direct future scientific and breeding programs.

**Methods:**

A comprehensive bibliometric analysis was conducted on a curated dataset of forty open-access research articles published between 2010 and 2024, sourced from Web of Science and Scopus. Following rigorous data deduplication, the bibliometrix R package was employed to analyze publication trends, collaboration networks, and keyword co-occurrence patterns. Collaboration networks were subsequently visualized and analyzed using VOSviewer.

**Results:**

The analysis identified a striking surge in annual publications, particularly post-2019, underscoring growing interest. China was the unequivocal dominant force in production and citation impact. Key research themes clustered around “fruit quality,” “genetics,” and “postharvest physiology,” with “quality” exhibiting the strongest network centrality. The network analysis identified “flat peach” as a central hub, demonstrating the highest co-occurrence link strength and affirming its strong, multifaceted connections within the research domain.

**Discussion:**

This first systematic bibliometric review delineates the rapid evolution of flat peach research, highlighting its core drivers and conceptual focus. The steep growth in output reflects rising academic and commercial interest. Critically, the analysis identifies significant gaps in molecular genomics, postharvest optimization, and climate resilience research. These findings provide a strategic, evidence-based roadmap to align future research priorities with market demands and sustainable cultivation challenges.

## 1 Introduction

Structural variation (SVs) represents a major class of genomic variation involving large-scale alterations such as deletions (>50 bp), duplications, inversions, translocations, and presence/absence variations (PAVs) (Yuan et al., 2021). Unlike single-nucleotide polymorphisms (SNPs) and small indels, SVs have a more pronounced impact on gene function, regulatory networks, and phenotypic diversity due to their ability to modify gene dosage, disrupt coding sequences, and alter chromatin architecture (Jayakodi et al., 2024; Kim et al., 2023; Zhang et al., 2024). While extensively studied in humans and livestock, where SVs are linked to numerous diseases (Aydin et al., 2025; Chen et al., 2024; Jiang et al., 2023; Pietryga et al., 2025; Yang et al., 2025; Zhou, 2024) and their role in plant genomes is significant. In crops, SVs are now recognized as pivotal drivers of domestication, adaptation, and agronomic trait variation (Wang et al., 2023; Jiao et al., 2025). Advances in long-read sequencing and pangenome analyses have revealed that SVs contribute more heritable phenotypic variation than SNPs in key traits such as flowering time, stress tolerance, and yield (Cui et al., 2025; Guan et al., 2021; Guo et al., 2020; Jayakodi et al., 2024; Jiao et al., 2025; Kim et al., 2023; Li et al., 2025; Wang et al., 2023; Zhang et al., 2024). For instance, SVs underlie the pod-shattering resistance gene PDH1 in soybean (affecting seed size) (Kobayashi et al., 2025) and reshape population gene expression traits (Zhang et al., 2024). Pangenome era, a systematic understanding of SVs will be essential for unlocking the full genetic potential of crops.

In modern plant breeding, SVs mapping is revolutionizing pedigree analysis (Fresnedo-Ramírez et al., 2016) by decoding the molecular basis of historical breeding selections, enabling precise tracking of beneficial alleles through breeding pedigrees, and revealing cryptic variation undetectable by SNP-based approaches (Zhou et al., 2016). Integrating SVs data with advanced breeding strategies, including genomic selection and gene editing, accelerates the development of climate-resilient cultivars. As we enter the pangenome era, a comprehensive understanding of SVs will be paramount for unlocking the full genetic potential of crop species. SVs have different types. The major types of structural variations are presence–absence variation, copy number variation, homoeologous exchange, deletion, duplication, insertion, inversion, and translocation (Cui et al., 2025; Guan et al., 2021; Yuan et al., 2021; Zhang et al., 2024). These SVs are associated with human and animal diseases and are central to plant diversity, adaptation, and breeding. Advanced technologies now enable their comprehensive detection and application in crop improvement (**Table 1**).

**Table 1:** Types and potential impact of SVs.

SV type	Definition	Potential impact	Reference
Presence–Absence Variation (PAV)	A DNA segment (gene or sequence) is present in some individuals but absent in others	Drives trait diversity, adaptation, and domestication by introducing or removing functional genes	(Li et al., 2025)
Copy Number Variation (CNV)	Variation in the number of copies of a particular DNA segment among individuals.	Alters gene dosage, affects gene expression, and can lead to new traits or stress responses	(Li et al., 2025)
Homoeologous Exchange (HE)	Replacement of a genomic segment by a similar copy from another genome or duplicated region (common in polyploids).	Generates novel gene combinations, impacts adaptation, and is frequent in polyploid crops	(Cui et al., 2024)
Deletion	Loss of a DNA segment from the genome.	Remove genes or regulatory elements, leading to loss of function or altered traits	(Guan et al., 2021; Li et al., 2025)
Duplication	Copying of a DNA segment, resulting in extra genetic material.	Increases gene dosage, can create new gene functions, and drives phenotypic diversity	(Tan et al., 2021)
Insertion	Addition of a DNA segment into the genome.	Introduces new genetic material, potentially creating novel traits or disrupting existing genes	(Li et al., 2025; Cao et al., 2014)
Inversion	A DNA segment is reversed end-to-end within the chromosome.	Alters gene order, can disrupt gene function, or regulation	(Guan et al., 2021; Guo et al., 2020)
Translocation	Rearrangement of DNA between non-homologous chromosomes.	Create new gene combinations, break genetic linkages, and impact trait inheritance	(Li et al., 2025)

Scholars investigated the major origin of SVs. Structural variations originate through multiple mechanisms, beginning with genome duplication and polyploidization, where whole-genome duplications (WGDs) and hybridization (auto- or allopolyploidy) generate large-scale genomic rearrangements (Gabur et al., 2019). A second major source is chromosomal instability, where errors during mitosis or meiosis lead to aneuploidy, dysploidy, and segmental rearrangements (Tian et al., 2021; De Storme and Mason, 2014). Additionally, transposable element (TE) activity drives SVs formation through insertions, deletions, and recombination, particularly in repetitive genomic regions (Hassan et al., 2024; Pinosio et al., 2016). Finally, exogenous factors, such as topoisomerase II inhibitors, can artificially induce SVs for experimental or breeding applications (Bechen et al., 2025). These mechanisms contribute to the dynamic genomic diversity observed across plant species.

Recent advances in sequencing technologies and computational methods have revolutionized the identification of structural variations (SVs) in plant genomes (Bansal and Boucher, 2019; Goodwin et al., 2016; Dossary et al., 2023; Eren et al., 2022; Amarasinghe et al., 2020). High-throughput whole-genome sequencing platforms, including both short-read (Illumina) and long-read technologies (PacBio, Oxford Nanopore), now enable comprehensive SVs detection through improved read lengths and alignment accuracy. These approaches leverage multiple detection strategies - including read-pair analysis, split-read mapping, and depth-of-coverage methods - to precisely identify diverse SV types ranging from small indels to complex chromosomal rearrangements (Aydin et al., 2025; Pietryga et al., 2025; Zhou, 2024).

Detecting SVs typically relies on two strategies. The first strategy entails a direct comparison of various genome assemblies. The second strategy involves using data from reads aligned to a reference genome, including paired reads (PR), read depth (RD), and split reads (SR), to identify SVs.

A variety of computational tools and algorithms have been developed to detect SVs from the sequencing data using the long-read and short-read sequencing platforms. For instance, tools such as BWA-MEM (https://github.com/lh3/bwa) and Bowtie2 (https://github.com/BenLangmead/bowtie2) align reads to a reference genome, while SVs callers such as Manta (https://github.com/Illumina/manta), Lumpy (https://github.com/arq5x/lumpy-sv), and Delly (https://github.com/dellytools/delly) integrate split-read and read-pair signals to call SVs from short-read sequencing data. Whereas for long-read sequencing data, aligners such as minimap2, NGMLR, pbmm2, and Winnowmap2 optimize mapping efficiency for noisy, lengthy reads, also callers such as Sniffles2 (https://github.com/fritzsedlazeck/Sniffles), cuteSV (https://github.com/tjiangHIT/cuteSV), svim (https://github.com/eldariont/svim), and PBSV (PacBio) (https://github.com/PacificBiosciences/pbsv) specialize in SVs identification. For population-level studies, merging tools such as SURVIVOR (https://github.com/fritzsedlazeck/SURVIVOR/wiki) and panpop (https://github.com/starskyzheng/panpop) play significant roles in improving reliability and comparisons. Whereas Annotation pipelines like ANNOVAR, SnpEff, and the Variant Effect Predictor (VEP) are some of the tools to annotate the SVs regions and their impacts on the specific phenotypic traits. The integration of multi-omics data also provides insights into SV mechanisms.

Interestingly, scholars’ findings revealed that SVs have a significant impact on plant genomics and phenotypes. The large-scale genomic alterations can influence gene expression, regulatory networks, and phenotypic diversity, making them essential for understanding plant biology. For instance, structural variations in key genes have been linked to fruit quality traits in peach, apple, and tomato (Cui et al., 2025; Guan et al., 2021; Guo et al., 2020; Li et al., 2025; Wang et al., 2023), providing valuable insights for breeding programs. Additionally, comparative genomic analyses across different plant species have uncovered conserved and lineage-specific SVs, shedding light on evolutionary mechanisms and speciation events (Jayakodi et al., 2024; Jiao et al., 2025; Kim et al., 2023; Kobayashi et al., 2025; Ma et al., 2024; Zhang et al., 2024). These findings highlight the potential of leveraging SVs for crop improvement and sustainable agriculture.

Generally, SVs contributed to the phenotypic variations and the evolutionary process. In phenotypic Variation, SVs contribute significantly to heritable traits in both plants and humans, influencing everything from disease susceptibility to agricultural productivity (Ayalew et al., 2024; Chen et al., 2024; Kobayashi et al., 2025; Li et al., 2025; Wang et al., 2023; Zhang et al., 2024). Specific SVs are linked with specific agronomic traits. For instance, 1.67 Mb inversion at chromosome six in peach is linked with fruit shape, 1.3–2 kb insertions in the promoter region of RsMYB1.1 on chromosome two are linked with skin color of radish taproot. Additionally, in apple, a noncoding RNA generated from a 209 bp insertion in the intron of the mitogen-activated protein kinase (MAPK)-encoding gene *MMK2* regulates gene expression and influences fruit coloration (Wang et al., 2023).

Beyond single-nucleotide polymorphisms, SVs are a critical driver of genetic diversity that directly influence agronomic traits by altering gene structure and regulation (Cui et al., 2024, 2025; Li et al., 2025). This functional impact makes SVs powerful tools for modern breeders. They serve as markers for selecting desirable alleles, including those lost during domestication (Jin et al., 2023), and help explain the genetic basis of hybrid vigor, guiding the development of superior hybrids in crops like maize (Li et al., 2021; Yang et al., 2019). Looking forward, SVs are central to the promise of precision breeding. Genome editing technologies allow scientists to precisely engineer SVs, such as creating targeted deletions in wheat to adjust flowering time (Gimenez et al., 2025). This approach accelerates crop improvement by creating genetic variation that is often indistinguishable from natural mutations, thus streamlining the path from lab to field.

**Figure 1:**
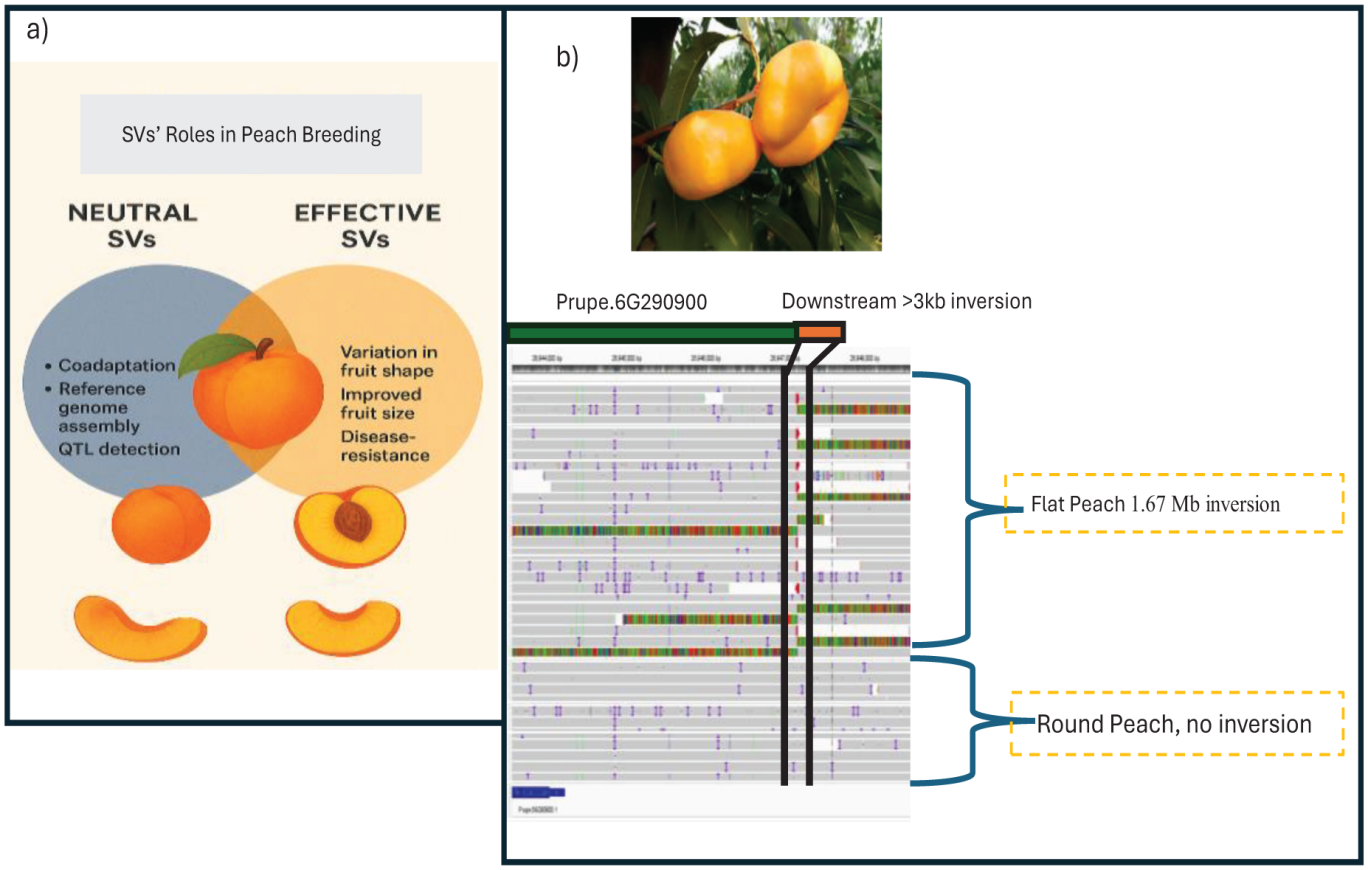
**(A)** Role of SVs in peach breeding. **(B)** Impact of SVs on Peach shape (A ~1.67 Mb inversion located ~3 kb downstream of Purpe. 6G290900 in a flat peach).

In peach breeding, SVs play a significant role, particularly those responsible for the shape of the peach (**Figures 1A, B**). Peaches and nectarines can be round or flat based on their shape. Flat peaches are called ‘Pan Tao’ and ‘Saturn peach’ or ‘donut peach’, respectively, in China and Western countries (Guo et al., 2018). The flat shape of peach fruits, known as ‘Pan Tao’ in Chinese, has been a subject of botanical interest in Western countries (Huang et al., 2008). Flat peach (*Prunus persica* L. Batsch. var. *compressa* Bean), originating from Xinjiang province, China, is a mutation of peach (*Prunus persica* L. Batsch) with a cultivation history of more than 2000 years (Gong et al., 2025). This unique type of peach was first introduced to Australia by the Chinese in the early nineteenth century and later brought to the USA in 1869, where it was referred to as the *Australian Saucer* (Okie et al., 1985). In the early 1950s, flat peaches were introduced to Sicily, Italy, where they continue to be cultivated in select niche regions (Cirilli and Rossini, 2021). The flat peach was cultivated in China two thousand years ago (Zhang et al., 2021). Flat-type peach and nectarine breeding activities have expanded globally since the 1980s (Cirilli and Rossini, 2021).

Researchers discovered and created markers that explain the differences in peach fruit shape. Fruit shape is associated with a 1.67 Mb inversion at chromosome six (Cao et al., 2016; Zhou et al., 2021) (**Figure 1**). The peach shape change is caused by a gene that is a member of the OVATE Family Protein PpOFP1 (Cao et al., 2016; Guan et al., 2021; Guo et al., 2020; Zhou et al., 2021). Fruit shape and size are controlled by cell division (cell number), distribution, and expansion (cell size) (Mauxion et al., 2021; Lopresti et al., 2014; Fresnedo-Ramírez et al., 2016). Flat peach cultivars had substantially fewer cells in the vertical axis because cell division in the vertical direction stopped early. The findings showed that the manner in which cells are produced in the vertical direction during the early stages of fruit development is the primary reason why peaches are either round or flat (Guo et al., 2018). In comparison to the round shape, flat peach varieties exhibit low titratable acidity, rich flavor, high sugar content, and a sweet taste (Veerappan et al., 2021). Consumers consider fruit shape an important external characteristic when selecting preferred fruit cultivars. To satisfy the consumers’ needs, in the last decade in China, many flat nectarine and peach varieties have been released and are under production. *Bian Tao* and *Sahuahong Pantao* are elite breeding material for flat Peach and nectarine breeding in China (Miao et al., 2022). Currently, many varieties of flat peach and flat nectarine are under production in China. For instance, Zhongpan 11, Zhongpan 13, Zhongpan 15, Zhongpan 17, Zhongpan 19, Zhongyoupan 7, Zhongyoupan 9, Jinxia Youpan, Jinxia Zaoyoupan, Zhongyoupanweimei, Zhongpan 101, and Zhongpan 102 are some of the improved flat peach and flat Nectarian varieties (Li and Wang, 2020; Miao et al., 2022; Pan et al., 2023; Zhu et al., 2020). Therefore, to satisfy peach consumers, it is crucial to comprehend the research progress on flat peaches and align the future research directions with the consumer needs. In this regard, Bibliometric analysis plays a significant role.

Bibliometric analysis is a powerful quantitative framework for mapping the evolution of scientific knowledge through large-scale evaluation of scholarly literature (Çelik, 2024; Visha et al., 2025). By leveraging computational algorithms and statistical metrics, this method systematically analyzes publication metadata, including citations, co-authorship networks, keyword co-occurrence, and temporal trends to uncover dominant research themes, transformative contributions, and emerging frontiers within a discipline (Shao, 2025; Qiliang Yang et al., 2023; Çelik, 2024; Visha et al., 2025; Ni et al., 2023). Unlike narrative reviews, bibliometrics provides a data-driven, reproducible approach to science mapping, minimizing selection bias while revealing macro-level patterns in research productivity, collaboration dynamics, and knowledge diffusion (Wallin, 2005). Its integration with machine learning and network theory further enhances predictive capabilities, enabling the identification of high-impact authors, institutional influences, and untapped interdisciplinary opportunities. This paradigm is increasingly adopted in evidence-based science policy and strategic research prioritization due to its objectivity and scalability (Uyanga et al., 2023).

Despite its utility, a comprehensive bibliometric synthesis has not been applied to the field of flat peach research, which has seen growing scientific and commercial interest in recent years. To address this gap, this study conducts a systematic bibliometric analysis to quantitatively and qualitatively evaluate the global research landscape over the past decade. We analyze key indicators, including publication trends, international collaborations, influential entities, and funding patterns, to map the intellectual structure and evolution of this field. This analysis is deliberately focused on open-access journal articles. While this methodological choice delimits the final corpus to 40 publications, it ensures full-text availability for consistent and reproducible data extraction, allowing for an in-depth analysis of the openly communicated scientific discourse. The findings aim to provide an evidence-based overview of major research fronts and emerging trends, serving as a foundational resource for the academic and industry community. Ultimately, this work seeks to identify critical knowledge gaps and guide strategic future research to advance both fundamental understanding and applied innovation in flat peach cultivation and utilization.

## 2 Methods

### 2.1 Data sources

The data for this study were obtained from the Web of Science Core Collections (WoSCC) and Scopus database, which are considered reliable and widely used for bibliometric research, using the advanced search mode (Shao, 2025; Visha et al., 2025). The publication period was only one decade set from 2010 to 2024, and the language was English and Chinese; the literature types were only “ARTICLE” and open access.

### 2.2 Search strategies

**Figure 2:**
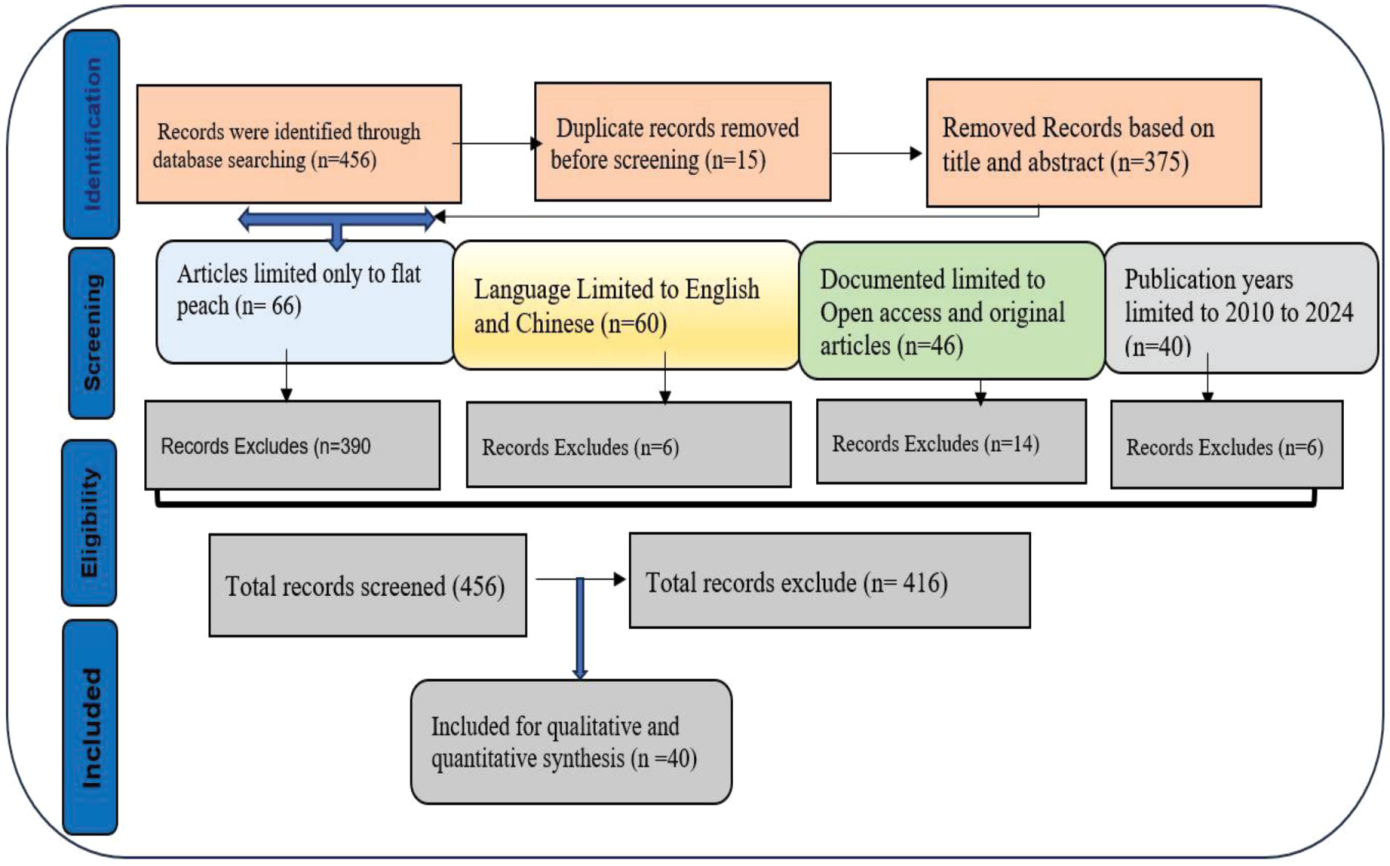
A PRISMA diagram showing the process involved for the selection of publications included in this study.

The Web of Science Core Collection and Scopus databases were searched using the keywords: “((peach) AND (flat))”. The search terms were applied to the titles, abstracts, and keywords. The Boolean operators “OR/AND” were used between keywords to search for relevant articles. A total of 456 (209 from Web of Science and 247 from Scopus database) results were retrieved, and the document types included research articles, review articles, proceeding papers, and editorial materials (**Figure 2**). A total of 40 literature were obtained after refining based on the setting criteria (**Figure 2**).

### 2.3 Data extraction

After successful searching, full records and citations of documents were exported from Web of Science and Scopus in a plain text format for further analysis. Data included information on authors, publication years, document types, Web of Science categories, affiliations, journals, funding agencies, countries, research areas, citations, and keywords.

### 2.4 Bibliometric and data analysis

First, we combined the two datasets from two databases using the Bibliometrix package in R. After combining, the detailed bibliometric analysis was conducted using the Biblioshiny under the Bibliometrix package in R (Aria and Cuccurullo, 2017). Results for primary publication data, annual scientific production and citations, significant sources, author impact, relevant affiliations, factorial analysis, collaboration index, and the most cited countries were generated. We employed R statistical software and Microsoft Excel for data analysis.

The Author Collaboration Index (ACI) and Collaboration Network were analyzed based on (Ajiferuke et al., 1988; Newman, 2003; Blondel et al., 2008) as follows: Equations 1, 2, respectively.

(1) $$ACI=\;\frac{AMADs\;}{MADs}$$

Where, ACI -Author Collaboration Index, AMAD -Authors of Multi-Authored Documents, MADs – Multi-Authored Documents.

(2) $$\text{Q}=\frac{1}{\text{d}}\sum_{\text{ij}}[{\text{a}}_{\text{ij}}-\frac{{\text{δ}}_{\text{i}}{\text{δ}}_{\text{ij}}}{2\text{h}}]{\text{s}}_{\text{i}}{\text{s}}_{\text{j}}$$

Where Q modularity score, δi indicates the degree of node i’ in the formula, h represents the total number of edges, and si represents the membership node to a community.

#### 2.4.1 Network analysis

For the analysis of research collaboration networks and knowledge structure, we adopted the comprehensive bibliometric network methodology used by (Hussain and Ali Bhat, 2023), employing co-authorship networks to map collaborative partnerships, keyword co-occurrence networks to identify conceptual domains, and citation networks to trace knowledge diffusion patterns, ensuring methodological consistency with contemporary bibliometric research standards.”

## 3 Results

### 3.1 Analysis of published literature

Through systematic searches in Web of Science and Scopus, we identified 40 original research articles focused exclusively on flat peach studies (**Table 2**). All selected publications were open-access, published between 2010 and 2024, with a notable increase in annual output after 2019. The analyzed literature involved 198 researchers, with an average of five authors per study (**Table 2**). To maintain focus, we excluded studies on round peach, ensuring our review specifically addressed flat peach-related research.

**Table 2:** Main characteristics of the published articles on flat peach from 2010 to 2024.

Description	Results
Timespan	2010:2024
Sources (Journals, Books, etc)	30
Documents	40
Annual Growth Rate %	8.16
Document Average Age	5.05
Average citations per doc	14.62
DOCUMENT CONTENTS	
Keywords Plus (ID)	224
Author’s Keywords (DE)	134
AUTHORS	
Authors	198
AUTHORS COLLABORATION	
Co-Authors per Doc	7.6
International co-authorships %	17.5
DOCUMENT TYPES	
Article	40

### 3.2 Publication trends

**Figure 3:**
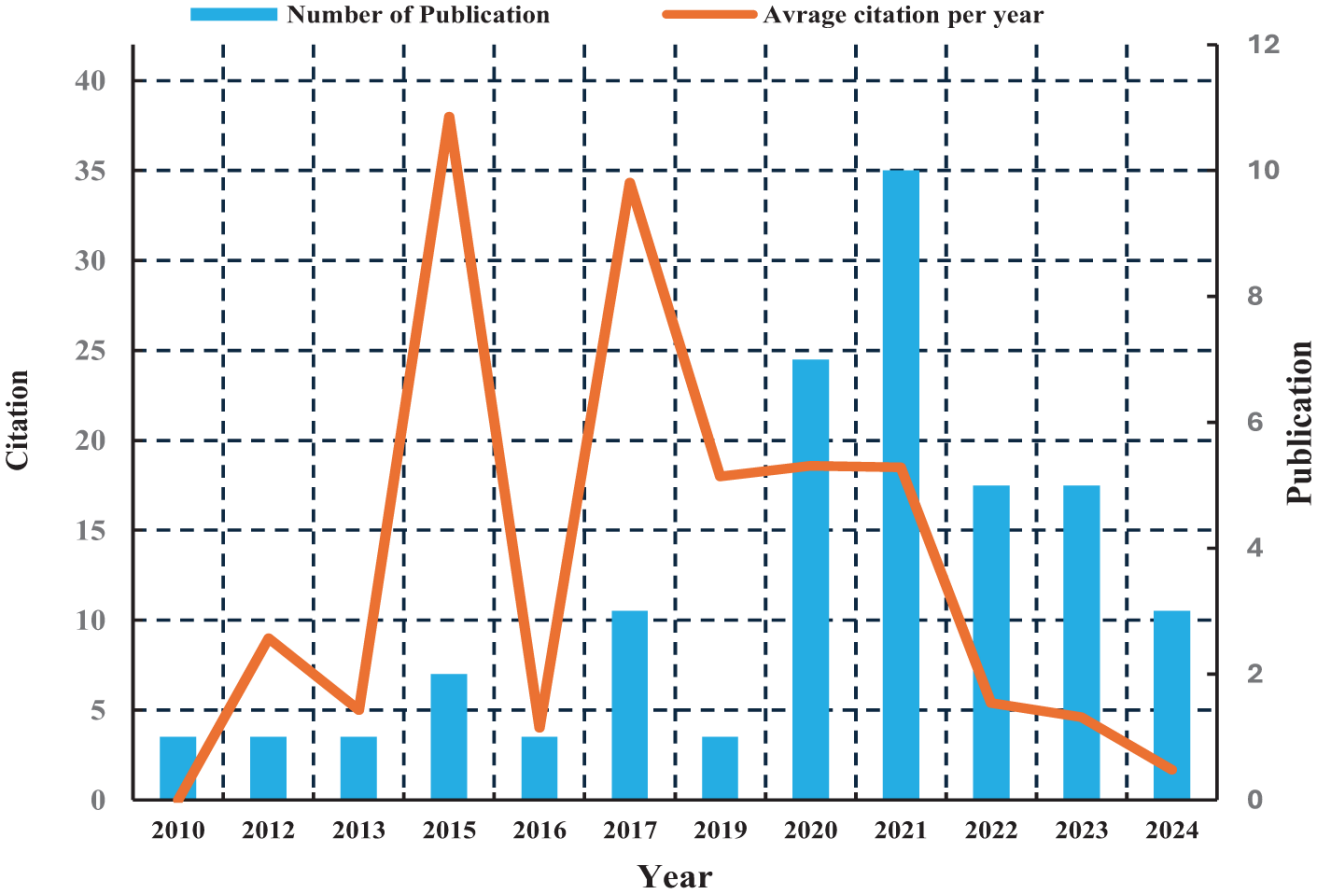
Annual production of publications and citations.

The number of publications on flat peach showed fluctuating growth between 2010 and 2024 (**Figure 3**). Only one article was published in 2010, followed by sporadic output in subsequent years, with no records in 2011, 2014, or 2018, and single articles in 2012, 2013, 2016, and 2019. Publication frequency increased notably after 2019, peaking in 2021 with 10 articles. Early publications received the highest citation counts, while annual output consistently surpassed previous years as research interest grew.

### 3.3 Sources document analysis

**Figure 4:**
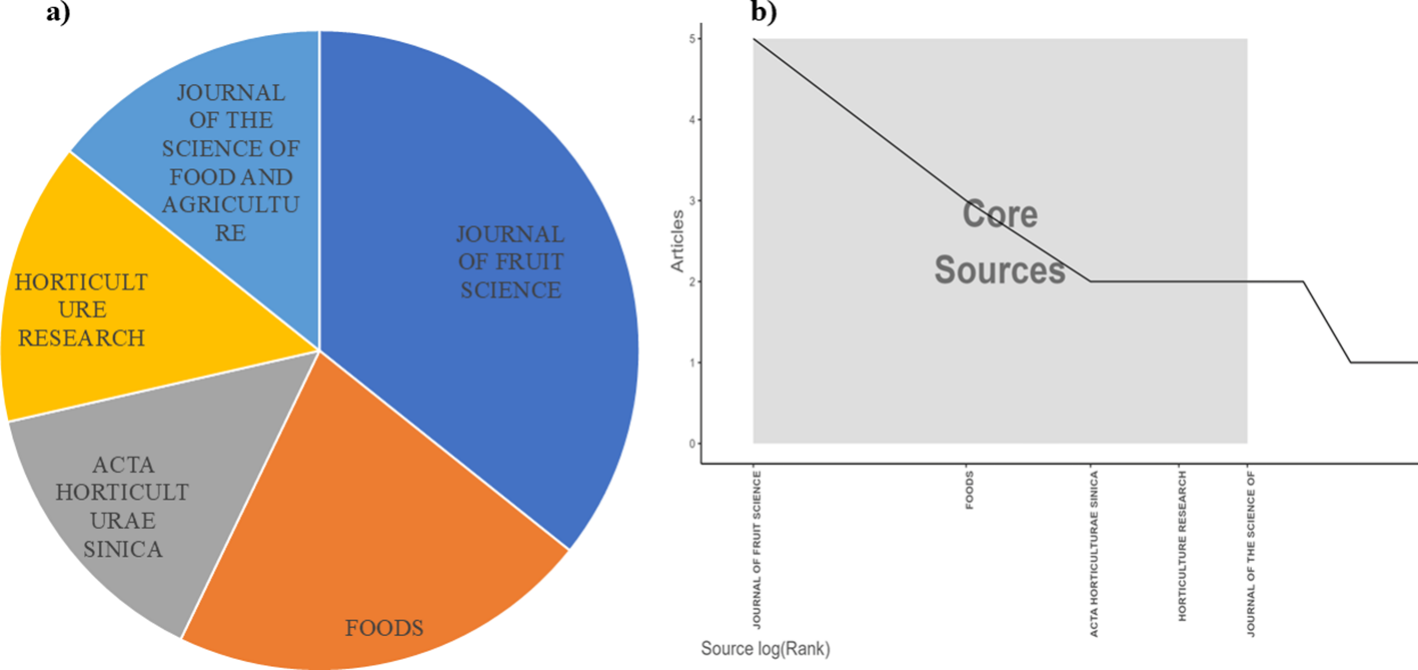
**(A)** Top five document sources **(B)** source score of articles based on Bradford’s Law.

Among the 40 publications analyzed, the Journal of Fruit Science emerged as the most active journal, publishing the highest number of studies on flat peach, followed by Foods (**Figure 4A**). Bradford’s Law of Scattering further validated the Journal of Fruit Science as the predominant core source for research in this field, demonstrating its central role in disseminating key findings (**Figure 4B**). The analysis also revealed that the citation more on plant biology, food science, and horticulture related journals (**Supplementary Table 1**, **Supplementary Figure 1**).

### 3.4 Author and institutional contributions

**Figure 5:**
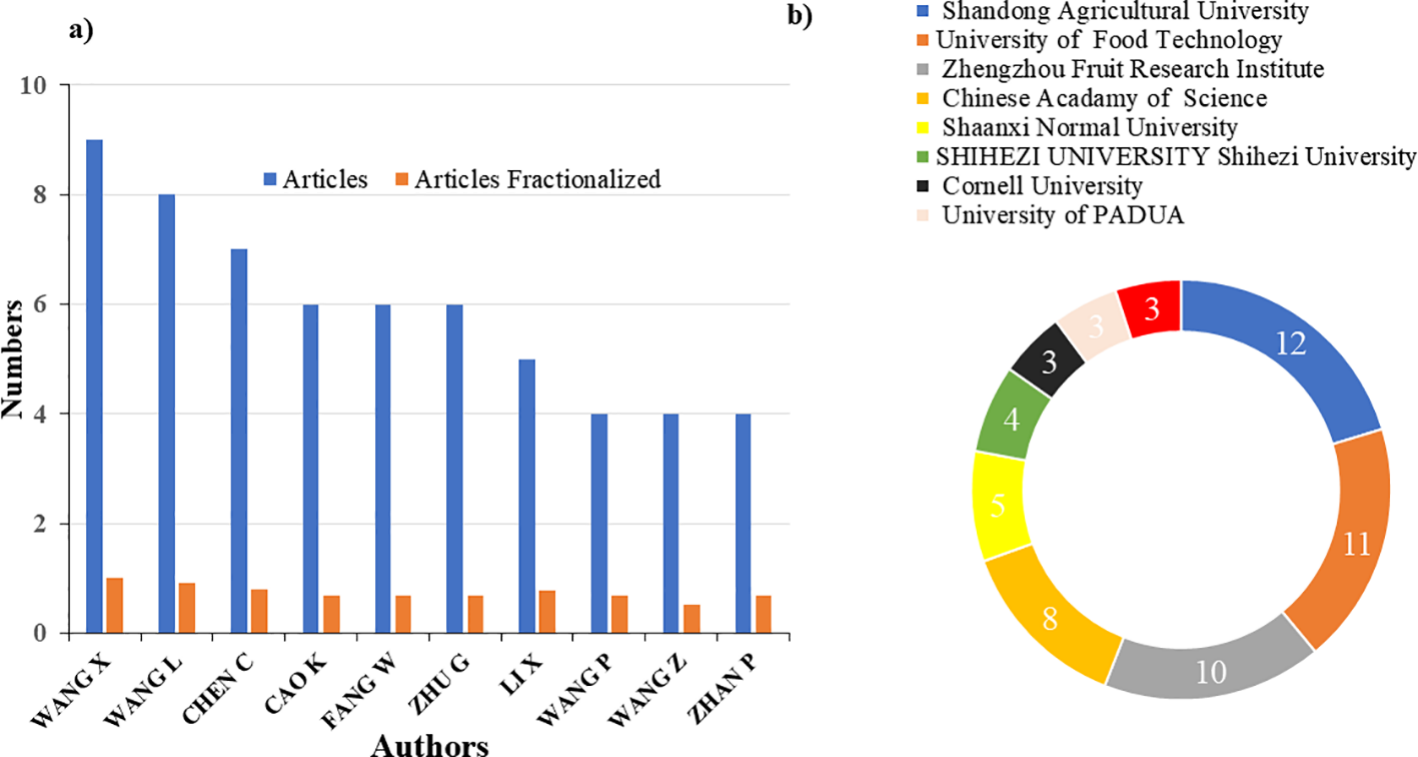
**(A)** Top ten authors (based on published articles and articles fractionalized). **(B)** The top ten affiliated institutes.

Our analysis revealed Wang X. as the most prolific author, with 9 publications, followed by Wang L., Chen C., and Cao K. as the top contributing authors (**Figure 5A**).

At the institutional level, Shandong Agricultural University had the highest representation, followed by the University of Food Technology, Zhengzhou Fruit Research Institute, and the Chinese Academy of Sciences as the leading contributing institutions (**Figure 5B**).

### 3.5 Country collaborations and cited information

**Figure 6:**
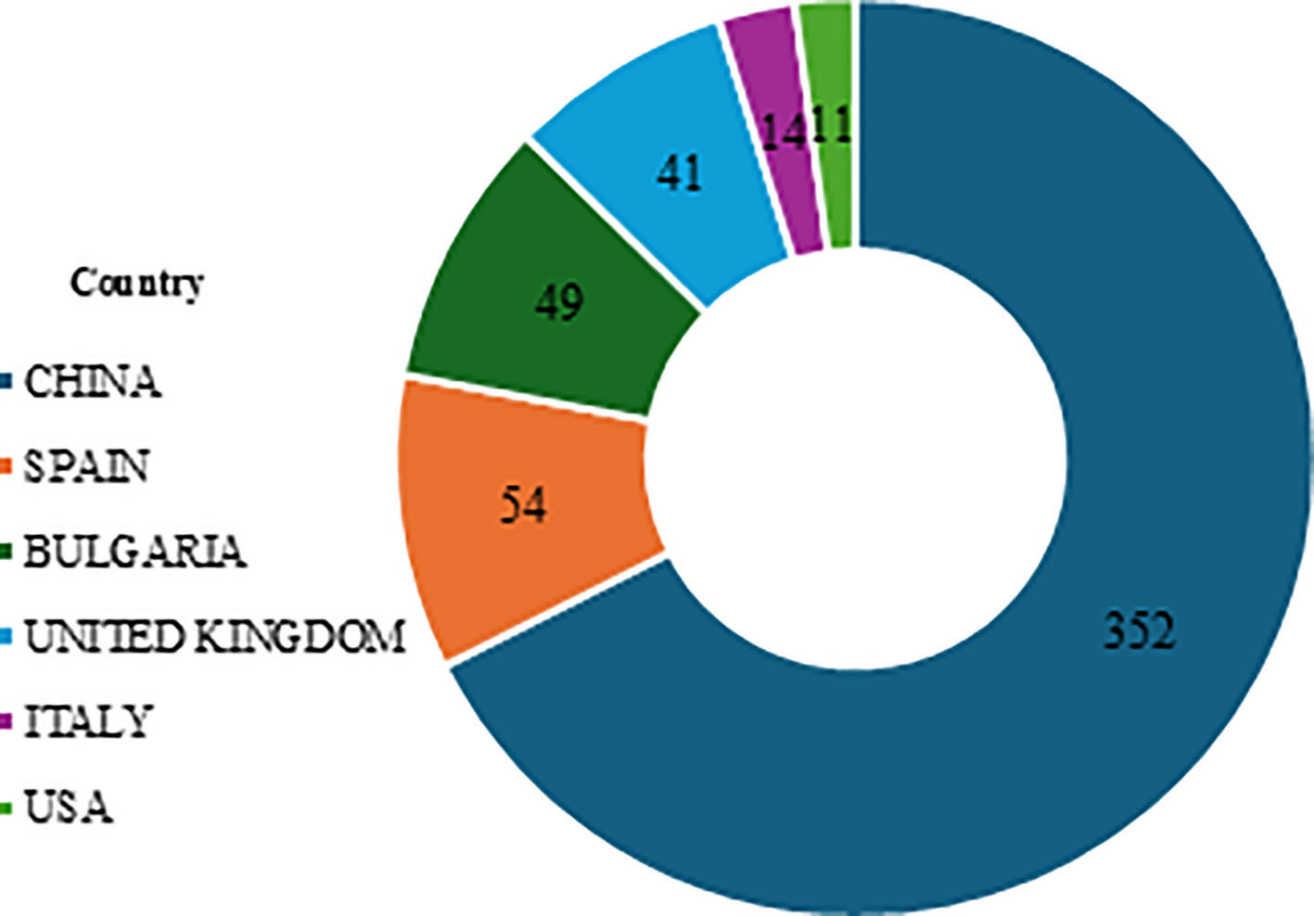
Country citations.

The collaboration and citation analysis revealed that China is the leading country in both collaboration and citations, followed by Spain, Bulgaria, the United Kingdom, Italy, and the USA (**Figure 6**).

### 3.6 Keyword frequency and trend analysis

**Figure 7:**
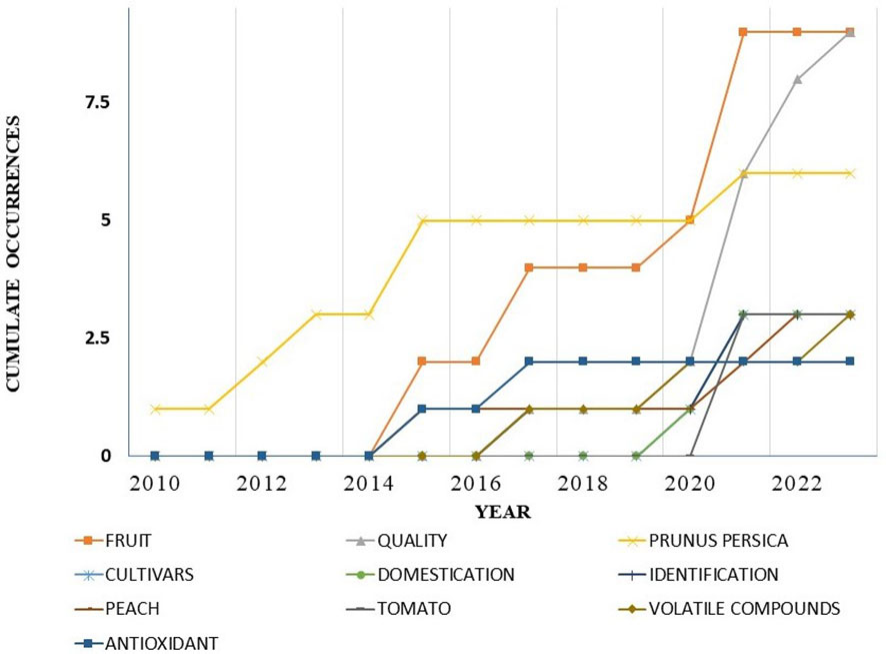
Most relevant words used in the study.

**Figure 8:**
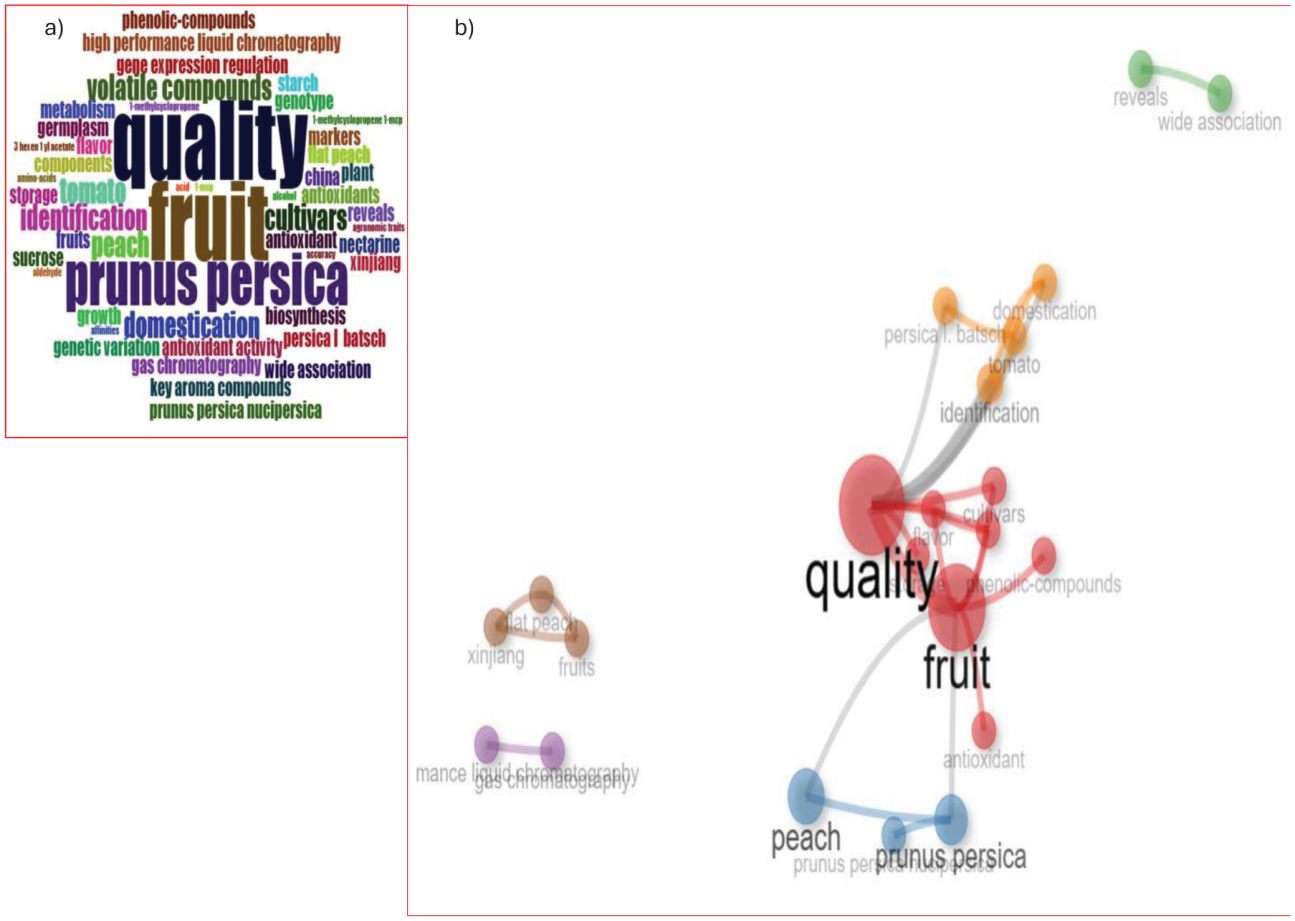
**(A)** Word frequency over time, **(B)** co-word network.

The analysis identified a set of unique keywords common to all reviewed publications. The five most frequent keywords were “fruit,” “quality,” “Prunus persica,” “cultivars,” and “domestication” (**Figure 7**). To assess temporal trends in keyword usage, a frequency-time analysis was performed. The results revealed a consistent linear increase in keyword occurrences over the study period. Notably, beginning in 2017, terms such as fruit, quality, and Prunus persica exhibited significant growth (**Figure 8A**). Among these, fruit appeared most frequently in recent years, highlighting its increasing importance in the field. The co-word network analysis revealed that quality exhibited the highest degree of connectivity with other keywords, indicating its central role in the thematic structure of flat peach research (**Figure 8B**).

### 3.7 A thematic and conceptual structure map

**Figure 9:**
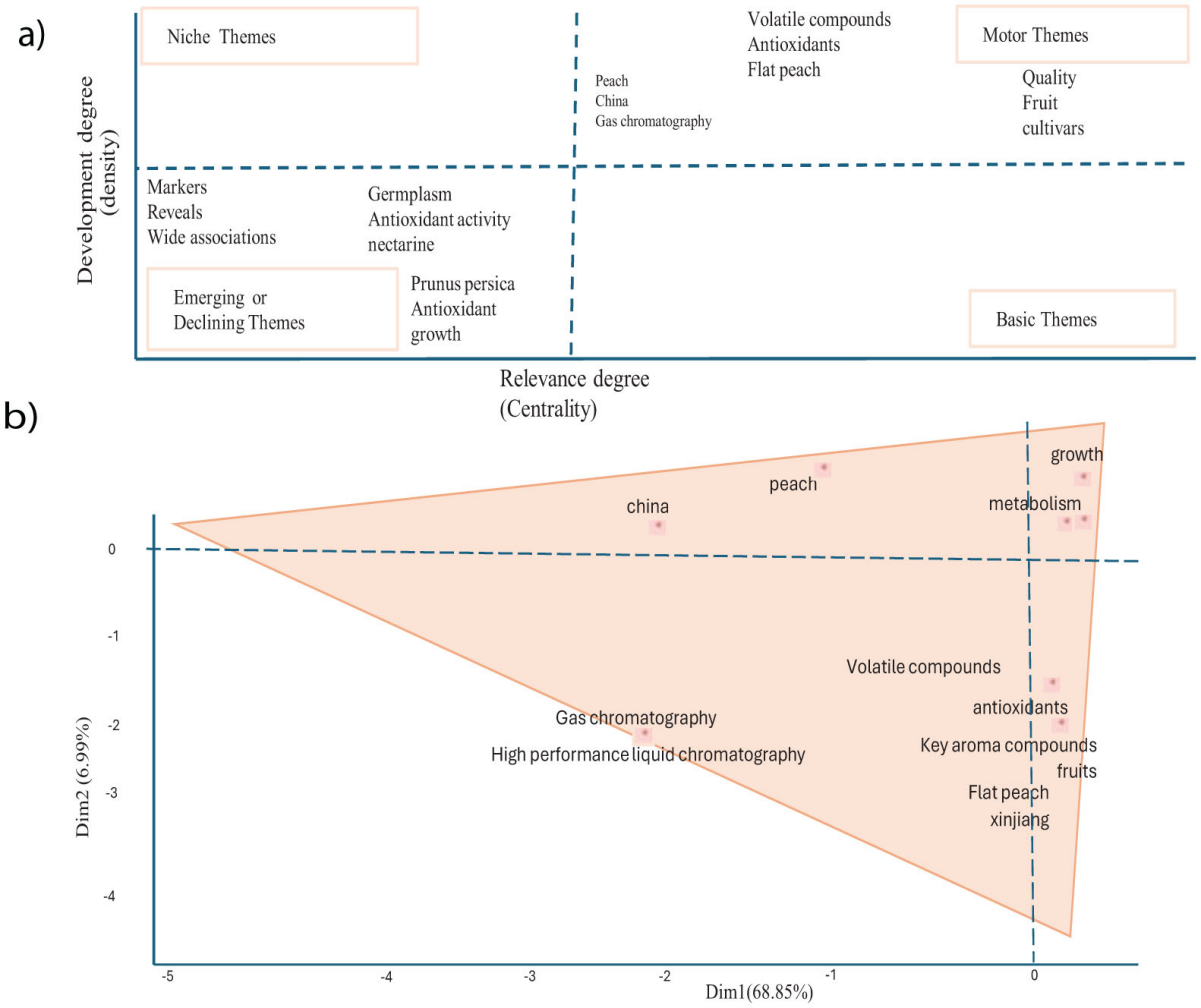
**(A)** Thematic maps of research themes and **(B)** a conceptual structure map.

A thematic map and conceptual structure map is a visualization tool used in bibliometric analysis to classify and represent the main research themes within a field based on keyword co-occurrence, citation networks, or text mining (**Figure 9A**). The result of thematic structure map shows that flat peaches, qualities, cultivars and other words under the motor themes it indicates that it’s well-developed, central topics driving the flat peach researchs. These are mature, active areas critical to the discipline (**Figure 9A**). The conceptual structural map showed that most of the teams contributed 68.85% dimensionality to the flat peach, Xinjiang fruits, and other teams on dim1 (**Figure 9B**).

### 3.8 Research collaboration networks and knowledge structure

This study employs a multidimensional network analysis to characterize the structure of flat peach research through co-authorship and co-occurrence. These perspectives collectively reveal the collaborative, conceptual, and intellectual dynamics of the field.

#### 3.8.1 Co-authorship network analysis

Co-authorship networks were constructed to visualize collaborative relationships among authors and their affiliated institutions.

##### 3.8.1.1 Author collaboration

**Figure 10:**
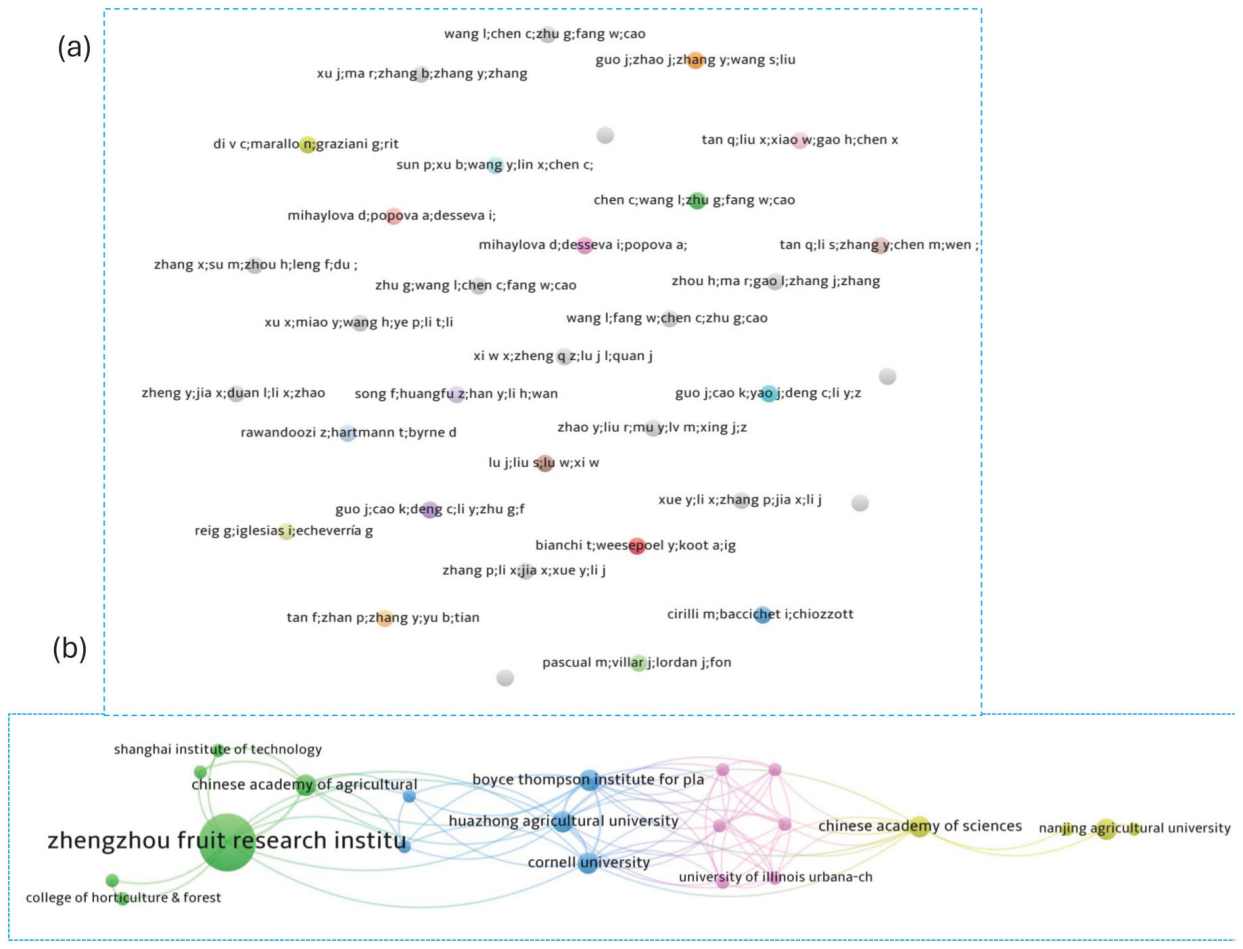
Co-authorship network analysis: **(A)** author collaboration network and **(B)** organizational collaboration network.

The author collaboration network, filtered to include authors with at least one document and one citation, comprised 39 individuals. Network analysis revealed a lack of significant collaborative clusters, indicating that co-authorship among these authors was limited and decentralized (**Figure 10A**).

##### 3.8.1.2 Organizational collaboration

Under the same publication and citation thresholds (≥1 document, ≥1 citation), the organizational network included all 78 institutions. The Zhengzhou Fruit Research Institute emerged as the most central organization, demonstrating the highest total link strength and underscoring its pivotal role within the collaborative network (**Figure 10B**).

#### 3.8.2 Network analysis of co-occurrences

The co-occurrence network analysis, synthesizing author and index keywords, elucidates a robust intellectual structure composed of distinct yet interconnected thematic clusters, revealing the field’s conceptual foundations and dynamic interfaces.

##### 3.8.2.1 Co-occurrence analysis in terms of all keywords

**Figure 11:**
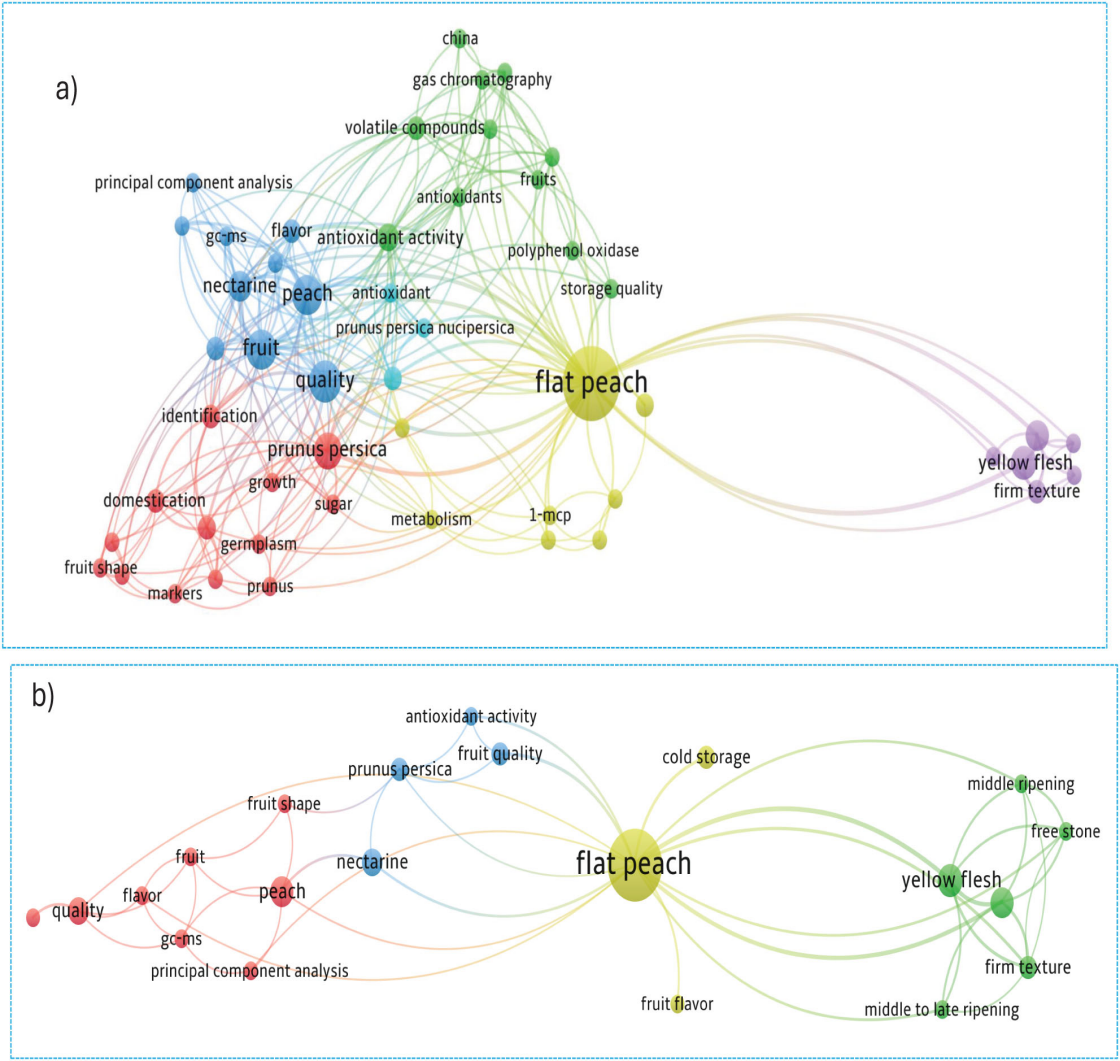
**(a)** Network analysis of all keywords and **(b)** authors' keywords co-occurrence.

The keyword co-occurrence network, delineated in **Figure 11A**, identified 51 key terms meeting the specified threshold, which collectively form a cohesive network structure. Analysis reveals six distinct thematic clusters, color-coded in the visualization, with node centrality indicative of frequency. The keyword “Flat Peach” emerged as the most prominent concept, demonstrating the highest occurrence frequency and total link strength, thereby signifying its pivotal role within the research landscape.

##### 3.8.2.2 Co-occurrence analysis in terms of author keywords

The co-occurrence analysis of author keywords, applying a minimum occurrence threshold of 2, identified 21 key terms from an initial set of 139 for network construction. These 21 keywords form a connected network visualized in **Figure 11B**, structured into four distinct clusters with a cumulative link strength of 170. The keyword “Flat peach” emerged as the most significant node, demonstrating the highest individual frequency (28 occurrences) and the greatest total co-occurrence link strength (37), indicating its central role within the research domain.

##### 3.8.2.3 Co-occurrence in terms of index keywords

**Figure 12:**
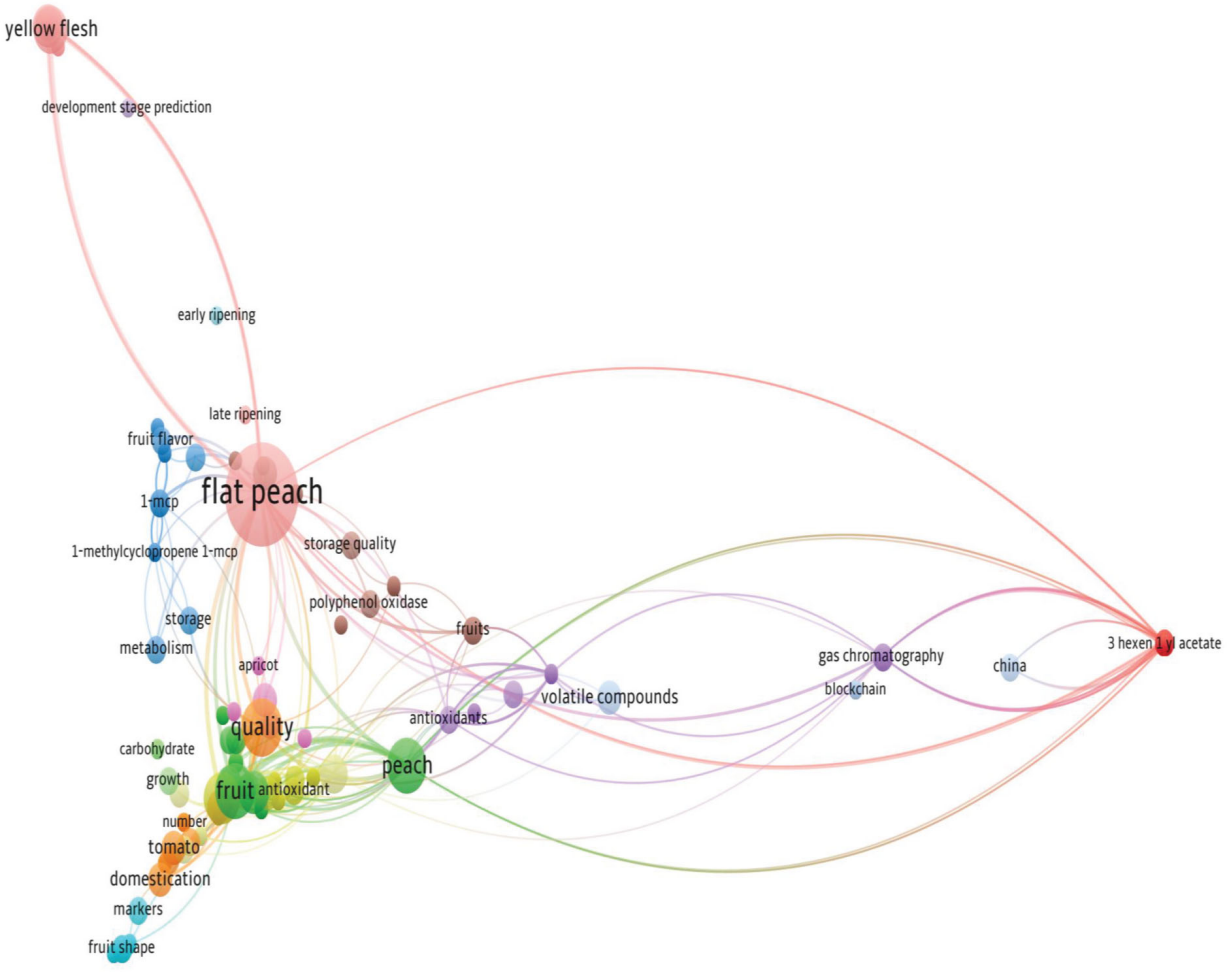
Network analysis of co-occurrence in terms of index keywords.

The co-occurrence analysis of index keywords, utilizing a minimum occurrence threshold of 2, identified 42 key terms from an initial pool of 259 for network mapping. These keywords form a structure of six clusters, visualized in **Figure 12**, where node size corresponds to frequency. The keyword “Flat Peach” was identified as the most influential, achieving the highest total link strength of 37 from 17 occurrences, confirming its pivotal role in the domain’s literature.

## 4 Discussion

This bibliometric analysis systematically maps the evolution of flat peach research, revealing distinct patterns in publication trends, geographic contributions, and thematic focus. The observed growth trajectory, particularly the accelerated output after 2019, reflects a rising academic and commercial interest in this type of peach. This surge likely stems from flat peaches’ documented sensory advantages, notably enhanced sweetness, flavor complexity, and reduced acidity, over conventional round peaches (Veerappan et al., 2021), traits that significantly influence consumer preference and market potential. The field’s development followed a characteristic innovation-adoption curve, with sporadic early publications (2010-2019) giving way to sustained research activity.

China’s dominance in both productivity and collaboration networks mirrors its strategic position in global peach production and agricultural R&D investment. Regional contributions from European countries with strong horticultural traditions, particularly Spain and Italy, highlight the crop’s importance in diverse growing environments. This geographic distribution suggests flat peach research is advancing through both centralized and specialized regional efforts.

Journal analysis identified a well-defined core-periphery structure, with the Journal of Fruit Science serving as the primary knowledge hub. The concentration of high-impact publications in agricultural and food science journals, validated by Bradford’s Law, indicates the field’s applied research orientation. This distribution provides clear guidance for future manuscript submissions and literature monitoring. The emergence of key research teams, particularly those affiliated with Shandong Agricultural University, demonstrates China’s capacity building in this domain. The citation patterns reveal an interesting dynamic: while foundational studies from 2010–2015 maintain high reference counts, recent work shows accelerated production rates, suggesting the field is transitioning from establishment to expansion phases.

Thematic evolution analysis uncovered three critical research dimensions: (1) fundamental fruit biology (Prunus persica), (2) quality attribute optimization, and (3) genetic improvement (cultivars, domestication). The centrality of quality in co-word networks underscores its role as a bridging concept between production and consumption studies. The temporal keyword shift toward holistic fruit characterization reflects maturation from component-based to systems-level research approaches.

## 5 Conclusions

We conducted the flat peach bibliometric analysis by highlighting the role of SVs as the major factors for the shape variations of peaches. This bibliometric analysis delineates the growth and thematic evolution of flat peach research, demonstrating its transition from a niche topic to an area of increasing scientific and commercial interest. China’s leading role, the emphasis on fruit quality, and the expanding publication trends all suggest that flat peaches are gaining recognition as a distinct and valuable crop. Future research should build on these foundations, particularly in genetics, postharvest, and market-driven studies, to further unlock the potential of this fruit.

## 6 Research frontiers and future directions

This bibliometric analysis has delineated the current intellectual structure of flat peach research, thereby revealing several critical knowledge gaps that present significant avenues for future investigation. To propel the field forward, research efforts must pivot towards addressing these key priorities. First, while breeding for enhanced organoleptic quality is a prominent theme, the molecular mechanisms governing these traits remain largely uncharacterized. A deeper genomic exploration, employing transcriptomic and proteomic approaches, is imperative to identify key genes and pathways responsible for fruit shape, flavor biosynthesis, and stress resilience, thereby accelerating precision breeding initiatives. Second, the high perishability of flat peaches remains a major barrier to market expansion (Gong et al., 2025), necessitating urgent innovation in postharvest physiology. Future work should focus on developing integrated, sustainable strategies, such as novel controlled atmosphere technologies and eco-friendly edible coatings, to extend shelf life without compromising the fruit’s distinct sensory profile. Third, despite documented sensory preferences (F. Tan et al., 2022), a robust, data-driven understanding of consumer behavior and market dynamics is conspicuously absent. Empirical studies utilizing econometric modeling and cross-cultural sensory analysis are essential to quantify commercial potential and inform supply chain strategies. Finally, given the escalating threats of anthropogenic climate change to crop productivity (Yadav et al., 2025), research into the abiotic stress tolerance of flat peaches is critically underdeveloped. Prioritizing the phenotyping of diverse germplasm for resilience to drought and heat, alongside the development of adaptive horticultural practices, is vital for ensuring the sustainable cultivation of this specialty crop in a changing global climate.

## Data Availability

The datasets presented in this study can be found in online repositories. The names of the repository/repositories and accession number(s) can be found in the article/**Supplementary Material**.

## Supplementary material

Supplementary Figure 1 Journal type and citations per year.
