# Supplementary material for: Research evolution of flat peach (Prunus persica (L.) Batsch): a decadal bibliometric analysis
Source: Front Plant Sci. 2025 Nov 28;16:1710142. doi: 10.3389/fpls.2025.1710142 (PMC12699350; doi:10.3389/fpls.2025.1710142)
Supplement: Supplementary file 1 [file Table1.docx]

**Supplementary Table2:** Details of documents bibliometrics

| **DOI** | **Authors** | **Title** | **Source** | **Year** | **TotalCitation** | **TCperYear** | **NTC** | **SR** | **quality** | **volatile compounds** | **prunus persica** | **peach** | **markers** | **antioxidant activity** | **Assigned_cluster** | **pagerank** |
| --- | --- | --- | --- | --- | --- | --- | --- | --- | --- | --- | --- | --- | --- | --- | --- | --- |
| 10.3390/foods10010164 | MIHAYLOVA D;POPOVA A;DESSEVA I;PETKOVA ;NADEZHDA N;STOYANOVA M;VRANCHEVA R;SLAVOV A;SLAVCHEV A;LANTE A | COMPARATIVE STUDY OF EARLY- AND MID-RIPENING PEACH (PRUNUS PERSICA L.) VARIETIES: BIOLOGICAL ACTIVITY, MACRO-, AND MICRO - NUTRIENT PROFILE | FOODS | 2021 | 32 | 6.4 | 1.73 | MIHAYLOVA D, 2021, FOODS | 0.323 | 0 | 0 | 0 | 0 | 0.677 | antioxidant activity | 0.039 |
| 10.3390/molecules26144183 | MIHAYLOVA D;DESSEVA I;POPOVA A;DINCHEVA ;IVAYLA I;VRANCHEVA R;LANTE A;KRASTANOV A | GC-MS METABOLIC PROFILE AND Α-GLUCOSIDASE-, Α-AMYLASE-, LIPASE-, AND ACETYLCHOLINESTERASE-INHIBITORY ACTIVITIES OF EIGHT PEACH VARIETIES | MOLECULES | 2021 | 17 | 3.4 | 0.919 | MIHAYLOVA D, 2021, MOLECULES | 0.203 | 0 | 0 | 0 | 0 | 0.797 | antioxidant activity | 0.052 |
| 10.21273/JASHS04990-20 | RAWANDOOZI Z;HARTMANN T;BYRNE D;CARPENEDO ;SILVIA S | HERITABILITY, CORRELATION, AND GENOTYPE BY ENVIRONMENT INTERACTION OF PHENOLOGICAL AND FRUIT QUALITY TRAITS IN PEACH | JOURNAL OF THE AMERICAN SOCIETY FOR HORTICULTURAL SCIENCE | 2021 | 11 | 2.2 | 0.595 | RAWANDOOZI Z, 2021, J AM SOC HORTIC SCI | 0 | 0 | 0 | 0 | 0 | 1 | antioxidant activity | 0.019 |
| 10.1186/s13059-020-02169-y | GUO J;CAO K;DENG C;LI Y;ZHU G;FANG W;CHEN C;WANG X;WU J;GUAN L;WU S;GUO W;YAO J;FEI ;ZHANGJUN Z;WANG L | AN INTEGRATED PEACH GENOME STRUCTURAL VARIATION MAP UNCOVERS GENES ASSOCIATED WITH FRUIT TRAITS | GENOME BIOLOGY | 2020 | 83 | 13.833 | 4.469 | GUO J, 2020, GENOME BIOL | 0.25 | 0 | 0 | 0 | 0.75 | 0 | markers | 0.022 |
| 10.3389/fpls.2019.00592 | TAN Q;LIU X;XIAO W;GAO H;CHEN X;FU X;LI L;LI D;GAO D | COMPARISON BETWEEN FLAT AND ROUND PEACHES, GENOMIC EVIDENCES OF HETEROZYGOSITY EVENTS | FRONTIERS IN PLANT SCIENCE | 2019 | 18 | 2.571 | 1 | TAN Q, 2019, FRONT PLANT SCI | 0 | 0 | 0 | 0 | 1 | 0 | markers | 0.02 |
| 10.1109/ACCESS.2020.2966020 | YU B;ZHAN P;LEI M;ZHOU F;WANG P | FOOD QUALITY MONITORING SYSTEM BASED ON SMART CONTRACTS AND EVALUATION MODELS | IEEE ACCESS | 2020 | 29 | 4.833 | 1.562 | YU B, 2020, IEEE ACCESS | 0 | 0.4 | 0 | 0.6 | 0 | 0 | peach | 0.016 |
| 10.3389/fmicb.2022.919047 | XU X;MIAO Y;WANG H;YE P;LI T;LI C;ZHAO R;WANG B;SHI X | A SNAPSHOT OF MICROBIAL SUCCESSION AND VOLATILE COMPOUND DYNAMICS IN FLAT PEACH WINE DURING SPONTANEOUS FERMENTATION | FRONTIERS IN MICROBIOLOGY | 2022 | 7 | 1.75 | 1.296 | XU X, 2022, FRONT MICROBIOL | 0 | 0 | 0 | 1 | 0 | 0 | peach | 0.035 |
| 10.1002/jsfa.6929 | DI V C;MARALLO N;GRAZIANI G;RITIENI A;DI M A | EVALUATION OF FRUIT QUALITY, BIOACTIVE COMPOUNDS AND TOTAL ANTIOXIDANT ACTIVITY OF FLAT PEACH CULTIVARS | JOURNAL OF THE SCIENCE OF FOOD AND AGRICULTURE | 2015 | 35 | 3.182 | 0.921 | DI V C, 2015, J SCI FOOD AGRIC | 0.113 | 0.198 | 0.556 | 0.132 | 0 | 0 | prunus persica | 0.078 |
| 10.3864/j.issn.0578-1752.2017.16.015 | LU J;LIU S;LU W;XI W | PHENOLIC PROFILES AND ANTIOXIDANT ACTIVITY OF FRUIT PULP FROM DIFFERENT TYPES OF PEACHES | SCIENTIA AGRICULTURA SINICA | 2017 | 9 | 1 | 0.262 | LU J, 2017, SCI AGRICULTURA SIN | 0.222 | 0 | 0.778 | 0 | 0 | 0 | prunus persica | 0.024 |
| 10.17660/actahortic.2012.962.76 | REIG G;IGLESIAS I;ECHEVERRÍA G | AGRONOMICAL PERFORMANCE, FRUIT QUALITY AND SENSORY ATTRIBUTES OF SEVERAL FLAT PEACH AND FLAT NECTARINE CULTIVARS | ACTA HORTICULTURAE | 2012 | 9 | 0.643 | 1 | REIG G, 2012, ACTA HORTIC | 0 | 0 | 1 | 0 | 0 | 0 | prunus persica | 0.011 |
| 10.1002/jsfa.6190 | PASCUAL M;VILLAR J;LORDAN J;FONSECA F;FALGUERA V;RUFAT J | RELATIONSHIP BETWEEN POLYPHENOL OXIDASE ACTIVITY AND NUTRITION, MATURITY AND QUALITY PARAMETERS IN FLAT PEACH | JOURNAL OF THE SCIENCE OF FOOD AND AGRICULTURE | 2013 | 5 | 0.385 | 1 | PASCUAL M, 2013, J SCI FOOD AGRIC | 0 | 0 | 1 | 0 | 0 | 0 | prunus persica | 0.008 |
| 10.1186/s12870-021-02850-9 | GUO J;CAO K;YAO J;DENG C;LI Y;ZHU G;FANG W;CHEN C;WANG X;WU J;GUO W;WANG L | REDUCED EXPRESSION OF A SUBUNIT GENE OF SUCROSE NON-FERMENTING 1 RELATED KINASE, PPSNRK1ΒΓ, CONFERS FLAT FRUIT ABORTION IN PEACH BY REGULATING SUGAR AND STARCH METABOLISM | BMC PLANT BIOLOGY | 2021 | 3 | 0.6 | 0.162 | GUO J, 2021, BMC PLANT BIOL | 0.157 | 0 | 0.66 | 0.183 | 0 | 0 | prunus persica | 0.069 |
| NA | HERNÁNDEZ F;MARTÍNEZ J;LEGUA P | AGRONOMIC AND QUALITY PARAMETERS OF FLAT PEACH UFO3, UFO4 AND SWEETCAP® | ITALIAN JOURNAL OF FOOD SCIENCE | 2010 | 0 | 0 | 0 | HERNÁNDEZ F, 2010, ITAL J FOOD SCI | 0 | 0 | 1 | 0 | 0 | 0 | prunus persica | 0.008 |
| 10.1016/j.foodres.2017.05.007 | BIANCHI T;WEESEPOEL Y;KOOT A;IGLESIAS ;IGNASI I;EDUARDO I;GRATACOS-CUBARSI M;GUERRERO L;HORTOS M;VAN R S | INVESTIGATION OF THE AROMA OF COMMERCIAL PEACH (PRUNUS PERSICA L. BATSCH) TYPES BY PROTON TRANSFER REACTION MASS SPECTROMETRY (PTR-MS) AND SENSORY ANALYSIS | FOOD RESEARCH INTERNATIONAL | 2017 | 49 | 5.444 | 1.427 | BIANCHI T, 2017, FOOD RES INT | 0.826 | 0.174 | 0 | 0 | 0 | 0 | quality | 0.067 |
| 10.1038/s41438-021-00648-2 | TAN Q;LI S;ZHANG Y;CHEN M;WEN ;BINBIN B;JIANG S;CHEN X;FU X;LI D;WU H;WANG Y;XIAO W;LI L | CHROMOSOME-LEVEL GENOME ASSEMBLIES OF FIVE PRUNUS SPECIES AND GENOME-WIDE ASSOCIATION STUDIES FOR KEY AGRONOMIC TRAITS IN PEACH | HORTICULTURE RESEARCH | 2021 | 38 | 7.6 | 2.054 | TAN Q, 2021, HORTIC RES-ENGLAND | 1 | 0 | 0 | 0 | 0 | 0 | quality | 0.051 |
| 10.1038/s41438-021-00661-5 | CIRILLI M;BACCICHET I;CHIOZZOTTO R;SILVESTRI ;CRISTIAN C;ROSSINI L;BASSI D | GENETIC AND PHENOTYPIC ANALYSES REVEAL MAJOR QUANTITATIVE LOCI ASSOCIATED TO FRUIT SIZE AND SHAPE TRAITS IN A NON-FLAT PEACH COLLECTION (P. PERSICA L. BATSCH) | HORTICULTURE RESEARCH | 2021 | 14 | 2.8 | 0.757 | CIRILLI M, 2021, HORTIC RES-ENGLAND | 0.743 | 0 | 0.257 | 0 | 0 | 0 | quality | 0.06 |
| 10.1016/j.lwt.2022.114388 | ZHANG X;SU M;ZHOU H;LENG F;DU ;JIHONG J;LI X;ZHANG M;HU Y;GAO Y;YE Z | EFFECT OF 1-METHYLCYCLOPROPENE ON FLAT PEACH FRUIT QUALITY BASED ON ELECTRONIC SENSES, LC-MS, AND HS-SPME-GC-MS DURING SHELF STORAGE | LWT-FOOD SCIENCE AND TECHNOLOGY | 2023 | 12 | 4 | 2.609 | ZHANG X, 2023, LWT-FOOD SCI TECHNOL | 1 | 0 | 0 | 0 | 0 | 0 | quality | 0.006 |
| 10.1111/jfpp.14575 | WANG P;TIAN H;TAN F;LIU Y;YU ;BIN B;ZHAN P | IMPACT OF COMMERCIAL PROCESSING ON VOLATILE COMPOUNDS AND SENSORY PROFILES OF FLAT PEACH JUICES BY PLSR AND BP NETWORK | JOURNAL OF FOOD PROCESSING AND PRESERVATION | 2020 | 8 | 1.333 | 0.431 | WANG P, 2020, J FOOD PROCESS PRESERV | 1 | 0 | 0 | 0 | 0 | 0 | quality | 0.065 |
| 10.3390/foods12193683 | ZHENG Y;JIA X;DUAN L;LI X;ZHAO ;ZHIYONG Z | SYNERGISTIC EFFECTS OF 1-MCP FUMIGATION AND Ε-POLY-L-LYSINE TREATMENTS ON DELAYING SOFTENING AND ENHANCING DISEASE RESISTANCE OF FLAT PEACH FRUIT | FOODS | 2023 | 1 | 0.333 | 0.217 | ZHENG Y, 2023, FOODS | 0.69 | 0 | 0.31 | 0 | 0 | 0 | quality | 0.04 |
| 10.3389/fnut.2022.965796 | SUN P;XU B;WANG Y;LIN X;CHEN C;ZHU J;JIA H;WANG X;SHEN J;FENG T | CHARACTERIZATION OF VOLATILE CONSTITUENTS AND ODOROUS COMPOUNDS IN PEACH (PRUNUS PERSICA L) FRUITS OF DIFFERENT VARIETIES BY GAS CHROMATOGRAPHY-ION MOBILITY SPECTROMETRY, GAS CHROMATOGRAPHY-MASS SPECTROMETRY, AND RELATIVE ODOR ACTIVITY VALUE | FRONTIERS IN NUTRITION | 2022 | 16 | 4 | 2.963 | SUN P, 2022, FRONT NUTR | 0.182 | 0.818 | 0 | 0 | 0 | 0 | volatile compounds | 0.025 |
| 10.1016/j.lwt.2023.114550 | ZHAO R;XU Y;LI C;WANG X;DU J;WANG C;SHI X;WANG B | ANALYSIS OF PHYSICOCHEMICAL CHARACTERISTICS, ANTIOXIDANT ACTIVITY, AND KEY AROMA COMPOUNDS OF FIVE FLAT PEACH CULTIVARS GROWN IN XINJIANG | LWT | 2023 | 9 | 3 | 1.957 | ZHAO R, 2023, LWT | 0 | 0.739 | 0 | 0.261 | 0 | 0 | volatile compounds | 0.056 |
| 10.7506/spkx1002-6630-20211011-107 | XIE X;LIU D;LI F;YAN Y;SONG F;CHEN J | EFFECT OF PROGRAMMED COOLING COUPLED WITH 1-METHYLCYCLOPROPENE OR PRESERVATIVE PAPER ON STORAGE QUALITY AND PEEL BROWNING OF FLAT PEACH GROWN IN XINJIANG; [程序降温协同1-甲基环丙烯, 保鲜纸两种处理对新疆蟠桃贮藏品质和果皮褐变的影响] | SHIPIN KEXUE/FOOD SCIENCE | 2022 | 0 | 0 | 0 | XIE X, 2022, SHIPIN KEXUE/FOOD SC | 0.069 | 0.931 | 0 | 0 | 0 | 0 | volatile compounds | 0.042 |
| 10.1111/pbi.13455 | ZHOU H;MA R;GAO L;ZHANG J;ZHANG A;ZHANG X;REN F;ZHANG W;LIAO L;YANG Q;XU S;OTIENO O C;ZHAO J;YU M;JIANG Q;KORBAN S;HAN ;YUEPENG Y | A 1.7-MB CHROMOSOMAL INVERSION DOWNSTREAM OF APPOFP1GENE IS RESPONSIBLE FOR FLAT FRUIT SHAPE IN PEACH | PLANT BIOTECHNOLOGY JOURNAL | 2021 | 49 | 9.8 | 2.649 | ZHOU H, 2021, PLANT BIOTECHNOL J | 0.486 | 0 | 0 | 0 | 0.257 | 0.257 |  | 0 |
| 10.1016/j.hpj.2017.01.012 | XI W X;ZHENG Q Z;LU J L;QUAN J Q | COMPARATIVE ANALYSIS OF THREE TYPES OF PEACHES: IDENTIFICATION OF THE KEY INDIVIDUAL CHARACTERISTIC FLAVOR COMPOUNDS BY INTEGRATING CONSUMERS' ACCEPTABILITY WITH FLAVOR QUALITY | HORTICULTURAL PLANT JOURNAL | 2017 | 45 | 5 | 1.311 | XI W X, 2017, HORTIC PLANT J | 0 | 0 | 0 | 0 | 0 | 0 |  | 0 |
| 10.1021/acs.jctc.5b00804 | PEACH M;TEALE A;HELGAKER T;TOZER D | FRACTIONAL ELECTRON LOSS IN APPROXIMATE DFT AND HARTREE-FOCK THEORY | JOURNAL OF CHEMICAL THEORY AND COMPUTATION | 2015 | 41 | 3.727 | 1.079 | PEACH M, 2015, J CHEM THEORY COMPUT | 0 | 0 | 0 | 0 | 0 | 0 |  | 0 |
| 10.3390/plants10030538 | ZHANG A;ZHOU H;JIANG X;HAN Y;ZHANG X | THE DRAFT GENOME OF A FLAT PEACH (PRUNUS PERSICA L. CV. ‘124 PAN’) PROVIDES INSIGHTS INTO ITS GOOD FRUIT FLAVOR TRAITS | PLANTS | 2021 | 20 | 4 | 1.081 | ZHANG A, 2021, PLANTS | 0 | 0 | 0 | 0 | 0 | 0 |  | 0 |
| 10.1016/j.foodcont.2023.110258 | SONG F;HUANGFU Z;HAN Y;LI H;WANG Z;JIN X;CHEN J | NITRIC OXIDE FUMIGATION CAN AFFECT THE METABOLISM OF VOLATILE COMPOUNDS DERIVED FROM ANALYSES OF FATTY ACIDS AND AMINO ACIDS IN POST-HARVEST FLAT PEACH DURING COLD STORAGE | FOOD CONTROL | 2024 | 5 | 2.5 | 3 | SONG F, 2024, FOOD CONTROL | 0 | 0 | 0 | 0 | 0 | 0 |  | 0 |
| 10.13925/j.cnki.gsxb.20190581 | WANG L;CHEN C;ZHU G;FANG W;CAO K;WANG X;WANG X;ZHAO P;WANG L | A NEW FLAT PEACH CULTIVAR 'ZHONGPAN 13'; [蟠桃新品种'中蟠13号'的选育] | JOURNAL OF FRUIT SCIENCE | 2020 | 5 | 0.833 | 0.269 | WANG L, 2020, J FRUIT SCI-a | 0 | 0 | 0 | 0 | 0 | 0 |  | 0 |
| 10.1590/fst.18022 | TAN F;ZHAN P;ZHANG Y;YU B;TIAN H;WANG P | DEVELOPMENT STAGE PREDICTION OF FLAT PEACH BY SVR MODEL BASED ON CHANGES IN CHARACTERISTIC TASTE ATTRIBUTES | FOOD SCIENCE AND TECHNOLOGY (BRAZIL) | 2022 | 4 | 1 | 0.741 | TAN F, 2022, FOOD SCI TECH | 0 | 0 | 0 | 0 | 0 | 0 |  | 0 |
| 10.13982/j.mfst.1673-9078.2016.5.039 | WANG P;TIAN H;ZHANG H;ZHAN P;TAN S;ZHANG H | DEVELOPMENT OF A CHROMATOGRAPHIC FINGERPRINT FOR FRESH FLAT PEACH BY GC-MS COMBINED WITH CHEMOMETRIC METHODS | MODERN FOOD SCIENCE AND TECHNOLOGY | 2016 | 4 | 0.4 | 1 | WANG P, 2016, MOD FOOD SCI TECHNOL | 0 | 0 | 0 | 0 | 0 | 0 |  | 0 |
| 10.13925/j.cnki.gsxb.20200040 | ZHU G;WANG L;CHEN C;FANG W;CAO K;WANG X;WANG J | A NEW FLAT PEACH CULTIVAR 'ZHONGPAN 17'; [中晚熟蟠桃新品种'中蟠17号'的选育] | JOURNAL OF FRUIT SCIENCE | 2020 | 3 | 0.5 | 0.162 | ZHU G, 2020, J FRUIT SCI | 0 | 0 | 0 | 0 | 0 | 0 |  | 0 |
| 10.13925/j.cnki.gsxb.20190635 | WANG L;FANG W;CHEN C;ZHU G;CAO K;WANG X;LI Y | A NEW MIDDLE RIPENING FLAT PEACH CULTIVAR 'ZHONGPAN 15'; [中熟蟠桃新品种'中蟠15号'的选育] | JOURNAL OF FRUIT SCIENCE | 2020 | 1 | 0.167 | 0.054 | WANG L, 2020, J FRUIT SCI | 0 | 0 | 0 | 0 | 0 | 0 |  | 0 |
| 10.21273/HORTSCI17046-22 | PAN L;NIU L;ZENG W;LU Z;CUI G;DUAN W;SUN S;WANG Z | ‘ZHONGPAN 101’ AND ‘ZHONGPAN 102’: TWO FLAT PEACH CULTIVARS FROM CHINA | HORTSCIENCE | 2023 | 1 | 0.333 | 0.217 | PAN L, 2023, HORTSCIENCE | 0 | 0 | 0 | 0 | 0 | 0 |  | 0 |
| 10.13925/j.cnki.gsxb.20200083 | CHEN C;WANG L;ZHU G;FANG W;CAO K;WANG X;NIU P | A NEW MIDDLE RIPENING FLAT PEACH CULTIVAR 'ZHONGPAN 19'; [中熟蟠桃新品种'中蟠19号'的选育] | JOURNAL OF FRUIT SCIENCE | 2020 | 1 | 0.167 | 0.054 | CHEN C, 2020, J FRUIT SCI | 0 | 0 | 0 | 0 | 0 | 0 |  | 0 |
| 10.16420/j.issn.0513-353x.2021-0085 | GUO J;ZHAO J;ZHANG Y;WANG S;LIU X;LI X;WANG Z;REN F;JIANG Q | A NEW LATE-RIPENING FLAT PEACH CULTIVAR 'RUIPAN 101' WITH YELLOW FLESH; [晚熟黄肉蟠桃新品种'瑞蟠 101 号'] | ACTA HORTICULTURAE SINICA | 2021 | 1 | 0.2 | 0.054 | GUO J, 2021, ACTA HORTIC SIN | 0 | 0 | 0 | 0 | 0 | 0 |  | 0 |
| 10.3390/foods13233835 | ZHAO Y;LIU R;MU Y;LV M;XING J;ZHENG L;AIHAITI A;WANG L | STUDY ON THE MECHANISMS OF FLAVOR COMPOUND CHANGES DURING THE LACTIC FERMENTATION PROCESS OF PEACH AND APRICOT MIXED JUICE | FOODS | 2024 | 0 | 0 | 0 | ZHAO Y, 2024, FOODS | 0 | 0 | 0 | 0 | 0 | 0 |  | 0 |
| 10.13982/j.mfst.1673-9078.2022.5.0959 | XUE Y;LI X;ZHANG P;JIA X;LI J | QUALITY VARIATIONS IN FLAT PEACHES WRAPPED IN DIFFERENT PLASTIC WRAPS DURING COLD STORAGE AND SHELF STORAGE AT ROOM TEMPERATURE; [不同保鲜膜包装蟠桃在冷藏和常温货架期间品质的变化] | MODERN FOOD SCIENCE AND TECHNOLOGY | 2022 | 0 | 0 | 0 | XUE Y, 2022, MOD FOOD SCI TECHNOL | 0 | 0 | 0 | 0 | 0 | 0 |  | 0 |
| 10.13995/j.cnki.11-1802/ts.029728 | ZHANG P;LI X;JIA X;XUE Y;LI J | EFFECTS OF MICRO-ENVIRONMENTAL MODIFIED ATMOSPHERE ON QUALITY AND PHYSIOLOGICAL CHANGES OF FLAT PEACH IN COLD STORAGE AND SHELF AT AMBIENT TEMPERATURE; [微环境气调对蟠桃冷藏和常温货架品质和生理变化的影响] | FOOD AND FERMENTATION INDUSTRIES | 2023 | 0 | 0 | 0 | ZHANG P, 2023, FOOD FERMENT IND | 0 | 0 | 0 | 0 | 0 | 0 |  | 0 |
| 10.16420/j.issn.0513-353x.2019-0806 | XU J;MA R;ZHANG B;ZHANG Y;ZHANG C;GUO L;SHEN Z;YU M | A NEW EARLY RIPENING RED-FLESH FLAT PEACH CULTIVAR‘JINLING XUEPAN’ | ACTA HORTICULTURAE SINICA | 2021 | 0 | 0 | 0 | XU J, 2021, ACTA HORTIC SIN | 0 | 0 | 0 | 0 | 0 | 0 |  | 0 |
| 10.13925/j.cnki.gsxb.20240019 | PAN L;NIU L;SUN S;DUAN W;CUI G;WANG Z;ZENG W | BREEDING REPORT OF A MID-LATE RIPENING YELLOW FLAT PEACH CULTIVAR ZHONGPAN 104; [中晚熟蟠桃新品种中蟠 104 的选育] | JOURNAL OF FRUIT SCIENCE | 2024 | 0 | 0 | 0 | PAN L, 2024, J FRUIT SCI | 0 | 0 | 0 | 0 | 0 | 0 |  | 0 |
