# Supplementary figures and images for: Research evolution of flat peach (Prunus persica (L.) Batsch): a decadal bibliometric analysis
Source: Front Plant Sci. 2025 Nov 28;16:1710142. doi: 10.3389/fpls.2025.1710142 (PMC12699350; doi:10.3389/fpls.2025.1710142)

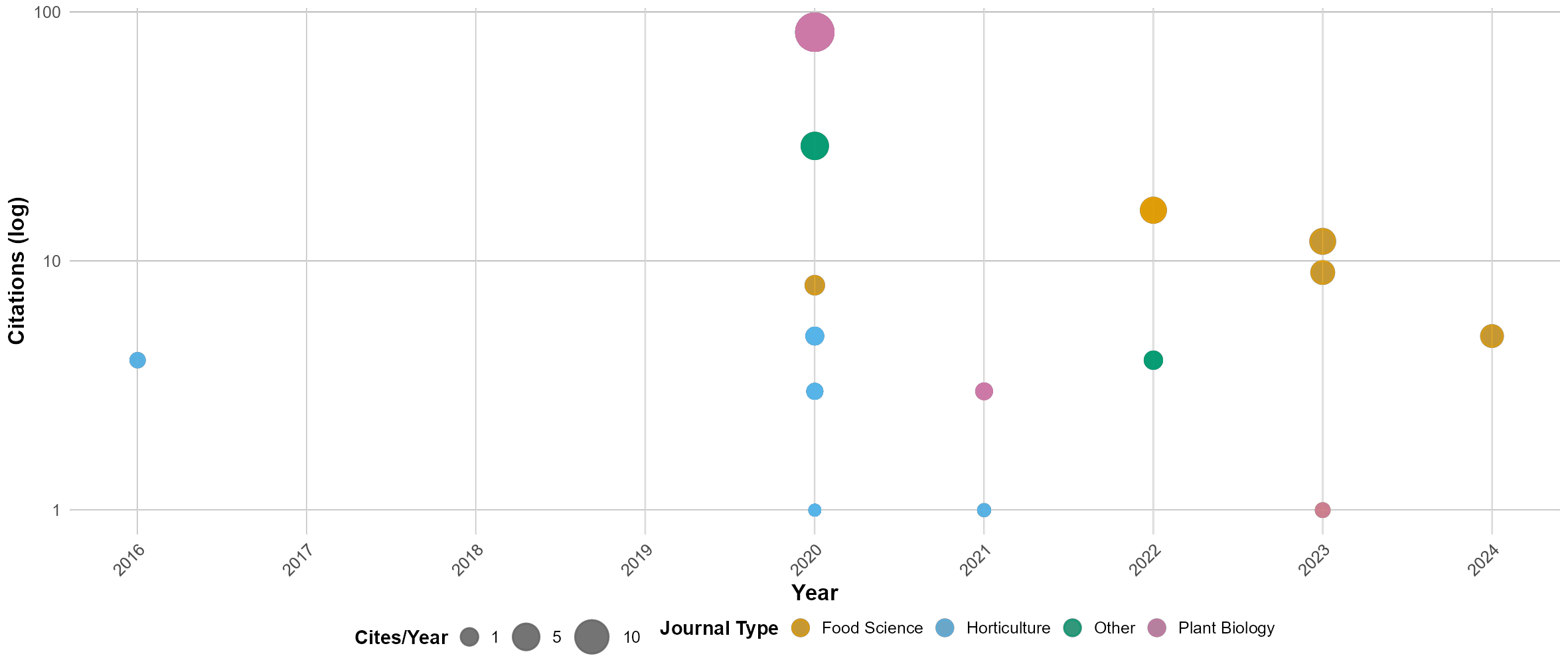

Supplement: Supplementary Figure 1 — Journal type and citations per year. [file Image1.tiff]
